# Functionalized Asymmetric Bola-Type Amphiphiles for Efficient Gene and Drug Delivery

**DOI:** 10.3390/nano8020115

**Published:** 2018-02-13

**Authors:** Zheng Huang, Dong-Mei Zhao, Xuan Deng, Ji Zhang, Yi-Mei Zhang, Xiao-Qi Yu

**Affiliations:** Key Laboratory of Green Chemistry & Technology (Ministry of Education), College of Chemistry, Sichuan University, Chengdu 610064, China; huangzheng1026@126.com (Z.H.); 2120170896@mail.nankai.edu.cn (D.-M.Z.); xiaohanchulin@pku.edu.cn (X.D.); ZYM12346@163.com (Y.-M.Z.)

**Keywords:** bolaamphiphiles, self-assembly, non-viral vector, gene delivery, drug delivery

## Abstract

The studies of bolaamphiphile-based nanoparticles as delivery vectors are still rudimentary and under development. In this study, several asymmetric bolaamphiphiles containing lysine and another moiety with special functions, such as pH-sensitive or cell-targeting property, were designed and synthesized. The potentials of these bolaamphiphile-based nanoparticles as versatile vectors for both nucleic acids and chemical drugs were studied. With the presence of 1,2-dioleoyl-*sn*-glycero-3-phosphoethanolamine (DOPE), these amphiphiles could be prepared into bolasomes, which showed good DNA binding ability and could condense plasmid DNA into nanoparticles with appropriate size and surface potential. **Lys-His**, which has a pH-sensitive histidine on one head, exhibited higher transfection efficiency than the symmetric counterpart and comparable efficiency to commercially available transfection reagent. Mechanism studies confirmed that the bolaplexes formed from **Lys-His** might induce the highest cellular uptake and the best endosomal escape ability. On the other hand, these bolaamphiphiles also exhibited good drug loading ability. The self-assembly vesicles could efficiently encapsulate the hydrophobic anti-cancer drug doxorubicin (DOX) in aqueous solution with high drug loading content and encapsulation efficiency. Confocal laser scanning microscopy (CLSM) experiment and cell viability assay exhibited a controlled release of the drug with the assistance of bolasomes. It was shown that such bolaamphiphiles have great potential as nano-vectors for both drug and gene or their co-delivery.

## 1. Introduction

Development of effective and safe delivery vectors is a critical challenge for the application of gene therapy, which provides a promising method for many acquired (such as AIDS or cancer) or inherited (cystic fibrosis etc.) diseases by transporting a specific gene to the target cells thus combating the disease at the level of its origin [1,2,3,4]. To date, although the use of viral vectors, including retroviruses and adenoviruses have been the most efficient strategy for gene therapy, some fatal disadvantages such as immunogenicity and potential insertional mutagenesis have obstructed their clinical applications [5,6]. Alternatively, a variety of synthetic (non-viral) delivery materials, including cationic lipids [7,8,9], polymers [10,11,12], dendrimers [13,14,15], and inorganic nanoparticles [16,17] are emerging as a promising tool for gene/drug delivery. Among them, cationic lipids have the widest applications because of their less immunogenic nature and the ease of handling and preparation [3,18,19].

However, unlike regular lipid-like amphiphiles (termed “mono-amphiphile”) consisting of one or more hydrophobic tails and a single hydrophilic headgroup, bola-type amphiphiles (termed “bolaamphiphile”), which are composed of one or two hydrophobic chains that are covalently linked at both ends to hydrophilic head groups, have not had extensive attention [20]. In nature, this type of dumbbell-shaped architecture has been mainly found in membranes of some certain kinds of extremophile archaebacteria [21]. Compared to the bilayer structures of normal lipids, the monolayer architecture is exceptionally stable and thus may ensure the survival of these bacteria in harsh environments [22,23,24,25]. Therefore, bola-type vectors are increasingly attractive for biomaterial applications ranging from nanomaterial synthesis to gene or drug delivery due to their unique molecular architecture. 

**Scheme 1:**
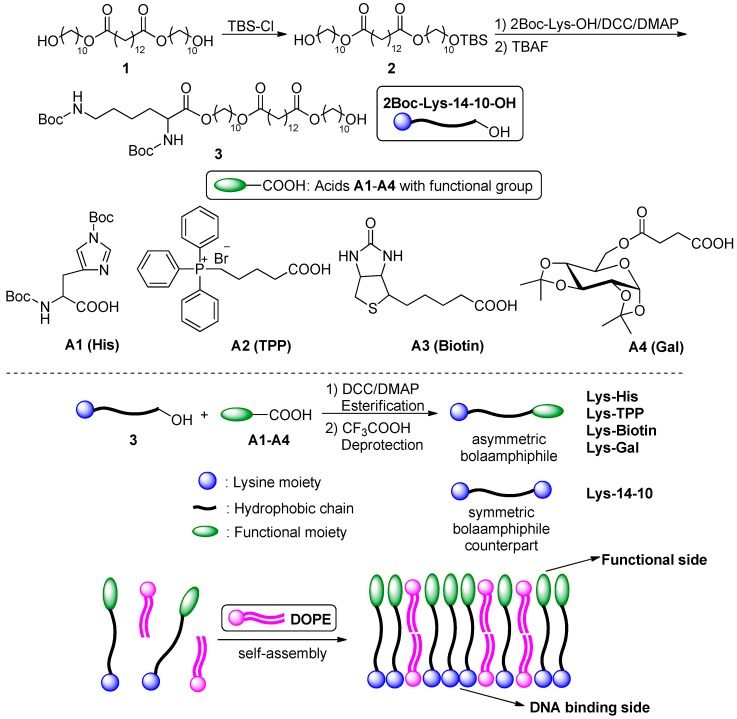
Synthetic routes of the target bolaamphiphiles.

We recently found that lysine or cyclen headgroups-based symmetric cationic bolalipids showed a certain potential for gene delivery [26]. However, due to the relatively low efficiency of cellular uptake and endosomal escape, the transfection efficiency (TE) was not satisfactory. Diverse strategies and methods were used to overcome these obstacles for obtaining effective bolaamphiphiles. Compared to the symmetric ones, asymmetric bolaamphiphiles have two different functional groups on each side. As a result, the positively charged side of the self-assembled bolasome may be used for DNA condensation, while another side may serve for pH-sensitive, targeting, or other functions (Scheme 1). It was reported that galactose-bearing asymmetric bolaamphiphiles could promote the gene transfection process by improving cellular uptake through ligand-receptor recognitions [22,27]. Specifically, galactose can selectively interact with asialoglycoprotein receptors (ASGPR) on the hepatocyte cell surface and then be rapidly internalized [28]. Biotin or biotin-conjugates are favorably integrated by tumor sites because of the overexpression of biotin-specific uptake systems on the cell surface in rapidly proliferating malignant cells [29]. Triphenylphosphine (TPP), which is a mitochondria-targeted group, also could be conjugated to two terminals of poly(ε-caprolactone) to form a bola-like structure for mitochondria-targeting drug delivery [30]. Furthermore, the pH sensitive group (imidazole) was also used to improve transfection performance of the bolaamphiphiles for siRNA delivery by facilitating endosomal escape through “proton sponge effect” [21,25]. 

Herein we report the rational design of asymmetric bolaamphiphiles functionalized with pH-buffering (histidine) or targeting (galactose, biotin, or TPP) groups. Their interaction with plasmid DNA and transfection mechanisms were systematically studied. Results revealed that these asymmetric bolaamphiphiles could give higher gene transfection efficiency than the symmetric counterpart. Moreover, the potential of these bolaamphiphile-based nanoparticles as nanocarriers for the anti-cancer drug doxorubicin (DOX) was preliminarily studied as well. It was shown that they could encapsulate the hydrophobic drug doxorubicin with a high drug loading content and encapsulation efficiency. 

## 2. Experimental Section

### 2.1. Materials and Methods

All of the common chemicals and reagents were obtained commercially and were used as received. Anhydrous chloroform and dichloromethane were dried and purified under nitrogen by using standard methods and were distilled immediately before use. Diol-14-10 (compound **1**), 2Boc-Histidine (**A1**) and the galactose derivative carboxylic acid (**A4**) were prepared according to the literature [26,31]. The (4-carboxybutyl)triphenylphosphonium bromide (**A2**) and biotin (**A3**) were purchased from Energy Chemical (Shanghai, China). pUC 19 DNA was purchased from TIANTAI (Chengdu, China). 1,2-dioleoyl-*sn*-glycero-3-phosphoethanolamine (DOPE) was purchased from Avanti Polar Lipids, Inc., Alabaster, AL, USA. Endotoxin-free plasmid purification kit was purchased from TIANGEN (Beijing, China). MicroBCA protein assay kit was obtained from Pierce (Rockford, IL, USA). Luciferase assay kit and MTS (3-(4,5-dimethylthiazol-2-yl)-5-(3-carboxymethoxyphenyl)-2-(4-sulfophenyl)-2H tetrazolium, inner salt) were purchased from Promega (Madison, WI, USA). The plasmids used in the study were pGL-3 (Promega, Madison, WI, USA) coding for luciferase and pEGFP-N1 (Clontech, Palo Alto, CA, USA) coding for EGFP. The Dulbecco’s modified Eagle’s medium (DMEM), opti-MEM culture medium, fetal bovine serum (FBS) and Lipofectamine^®^ 2000 were purchased from Invitrogen Corp (Carlsbad, CA, USA). The ^1^H NMR and ^13^C NMR spectra were measured on a Bruker AM400 NMR spectrometer. Proton Chemical shifts of NMR spectra were given in ppm relative to internals reference TMS (1H, 0.00 ppm). High resolution mass spectrometry (HRMS) spectral data was recorded on a Bruker Daltonics BioTOF mass spectrometer (Bruker Corporation, Billerica, MA, USA). 

Some procedures such as ethidium bromide replacement assay, dynamic light scattering (DLS) experiments (Malvern, Worcestershire, UK), transmission electron microscopy (TEM) (Hitachi High-Tech Science, Schaumburg, IL, USA), acid-base titration (PHS-300, Jiangsu, China), amplification, and purification of plasmid DNA, cell culture were performed according to the reported literature [26,32,33].

### 2.2. Synthesis of the Bolaamphiphiles

#### 2.2.1. Preparation of Compound **2**

Compound **1** (11.54 g, 20 mmol) and imidazole (1.28 g, 20 mmol) were dissolved in dry chloroform, a solution of *tert*-butyldimethylsilyl chloride (TBS-Cl, 2.40 g, 16 mmol) in anhydrous chloroform (80 mL) was added dropwise under an ice bath. The obtained mixture was then stirred at room temperature overnight. After dilution with Et_2_O (120 mL), the solution was washed with water (3 × 80 mL), dried over anhydrous Na_2_SO_4_, filtered and evaporated under reduced pressure. The residue was purified by silica gel column chromatography (PE:EA = 4:1, *v*/*v*) to give the product **2** as a colorless liquid. 

#### 2.2.2. Preparation of Compound **3**

2Boc-Lysine (11.8 mmol) was activated in the presence of dicyclohexylcarbodiimide (DCC, 2.44 g, 11.8 mmol) and *N*, *N*-dimethylaminopyridine (DMAP, 0.10 g, 0.79 mmol) in 120 mL anhydrous CH_2_Cl_2_ in an ice-salt-bath at 0 °C for 30 min. Then product **2** (11.8 mmol) was added and the mixture was stirred at room temperature overnight. The DCC condensation mixture was then filtered, and the filtrate was evaporated under reduced pressure, and a small amount of ethyl acetate was added. The mixture was maintained at 0 °C for half an hour and then filtered. The filtrate was evaporated under reduced pressure to give the crude product which was purified by column chromatography over silica gel (PE:EA = 2:1, *v*/*v*).

Subsequently, 1 M tetrabutylammonium fluoride (TBAF) THF solution was added to the anhydrous THF solution of the previously obtained precursor to make the final concentration of TBAF of 0.2 M and then stirred at room temperature for 6 h. The mixture was concentrated to afford an oil, which was further purified by column chromatography on silica gel (PE:EA = 2:1, *v*/*v*) to yield **3** as a white powder.

#### 2.2.3. Preparation of the Target Bolaamphiphiles

The target bolaamphiphiles were prepared by esterification and subsequent deprotection. Concretely, compound **3** (1.0 mmol) was respectively added to the anhydrous dichloromethane solution (60 mL) containing four kinds of acids (**A1**–**A4**, 1.2 mmol), DCC (247.4 mg, 2.2 mmol), and DMAP (12.2 mg, 0.1 mmol) in an ice-salt-bath at 0 °C for 1 h and then stirred at room temperature overnight. The DCC condensation mixture was then filtered, and the filtrate was evaporated under reduced pressure, and a small amount of ethyl acetate was added. The mixture was maintained at 0 °C for half an hour and filtered. The filtrate was evaporated under reduced pressure to give the crude products which were purified by column chromatography over silica gel (PE:EA = 1:1, *v*/*v*) to yield a white solid.

The target bolaamphiphiles were prepared by esterification and subsequent deprotection. Concretely, compound **3** (1.0 mmol) was respectively added to the anhydrous dichloromethane solution (60 mL) containing four kinds of acids (**A1**–**A4**, 1.2 mmol), DCC (247.4 mg, 2.2 mmol), and DMAP (12.2 mg, 0.1 mmol) in an ice-salt-bath at 0 °C for 1 h and then stirred at room temperature overnight. The DCC condensation mixture was then filtered, and the filtrate was evaporated under reduced pressure, and a small amount of ethyl acetate was added. The mixture was maintained at 0 °C for half an hour and filtered. The filtrate was evaporated under reduced pressure to give the crude products which were purified by column chromatography over silica gel (PE:EA = 1:1, *v*/*v*) to yield a white solid.

The previously obtained precursors (0.5 mmol) were dissolved in anhydrous dichloromethane (5 mL), and then, a solution of trifluoroacetic acid (5 mL) in anhydrous dichloromethane (5 mL) was added dropwise under an ice bath. Then, the obtained mixture was stirred at room temperature for 6 h. After the solvent and trifluoroacetic acid were removed, the target bolaamphiphiles were directly obtained as a flavescent ropy liquid by treating the residues twice with anhydrous ethyl ether. 

The detailed analysis data of the above compounds are listed in the supplementary files.

### 2.3. Formation of Bolasomes and Bolaplexes

Bolaamphiphiles (0.0025 mmol) and DOPE with mole ratio of 1:1 were dissolved in anhydrous chloroform (2.5 mL) in an autoclaved glass vial. Thin films were made by slowly rotary-evaporating the solvent at room temperature. The last trace of organic solvent was removed by keeping these films under vacuum above 8 h. The dried films and 2.5 mL deionized water were preheated to 70 °C, and then buffer was added to the films resulting in the final bola concentration of 1.0 mM. The mixtures were vortexed vigorously until the films were completely resuspended. Sonication of these suspensions for 5 min in a bath sonicator at 0 °C afforded the corresponding cationic bolasomes, which were then stored at 4 °C.

To prepare the bolaplexes, various amounts of bolasomes were mixed with a constant amount of DNA by pipetting thoroughly at various N/P ratios, and the mixture was incubated for 30 min at room temperature. The theoretical N/P ratio represents the charge ratio of cationic bolalipids to nucleotide base (in mole) and was calculated by considering the average nucleotide mass of 309.

### 2.4. Gel Retardation Assay

To determine the formation of bola/pDNA complex (bolaplexes), bolaplexes of various N/P ratios ranging from 0 to 8 were prepared as described above. A constant amount of 0.125 μg DNA was used here; 15 μL of each bolaplexes solution was electrophoresed on the 1% (*w*/*v*) agarose gel containing Gel-Red and Tris-acetate (TAE) running buffer at 135 V for 30 min. DNA was visualized with a UV lamp using a BioRad Universal Hood II.

### 2.5. In Vitro Transfection Procedure

In order to obtain about 80% confluent cultures at the time of transfection, 24-well plates were seeded with 1.0 × 10^5^ cell/well in 500 μL of antibiotic-free media 24 h before transfection. For the preparation of bolaplexes applied to cells, various amounts of bolasomes and DNA were serially diluted separately in opti-DMEM culture medium; then, the DNA solutions (50 μL) were added into the bolasome solutions (50 μL) and mixed briefly by pipetting up and down several times. After which the mixtures were incubated at room temperature for about 30 min to obtain bolaplexes of the desired N/P ratios, the final bolaplexes volume was 100 μL, and the DNA was used at a concentration of 0.8 μg/well. After 30 min of complexation, old cell culture medium was removed from the wells, cells were washed once with serum-free DMEM, and the above 100 μL bolaplexes were added to each well. The plates were then incubated for 4 h at 37 °C in a humidified atmosphere containing 5% CO_2_. At the end of the incubation period, medium was removed, and 500 μL of fresh DMEM medium containing 10% FBS was added to each well. Plates were further incubated for a period of 24 h before checking the reporter gene expression.

For fluorescent microscopy assays, cells were transfected by complexes containing pEGFP-N1. After 24 h incubation, microscopy images were obtained at a magnification of 100 and recorded using Viewfinder Lite (1.0) software. Control transfection was performed in each case using a commercially available transfection reagent Lipofectamine 2000^TM^ based on the standard conditions specified by the manufacture. After 24 h transfection of pEGFP plasmid, the eGFP expression was observed by an inverted fluorescent microscope. Excitation wavelength was 485 nm and the emission wavelength was 538 nm. Lipofectamine 2000^TM^ was chosen as control.

For luciferase assays, cells were transfected by complexes containing pGL-3. For a typical assay in a 24-well plate, 24 h post transfection as described above, the old medium was removed from the wells, and the cells were washed twice with 500 μL of prechilled phosphate buffered saline (PBS). According to Luciferase assay kit (Promega) manufacture, 100 μL of 1 × cell lysis buffer diluted with PBS was then added to each well, and the cells were lysed for 30 min in a horizontal rocker at room temperature. The cell lysate was transferred completely to Eppendorf tubes and centrifuged (12,000 rpm, room temperature (RT)) for 10 min; the supernatant was transferred to Eppendorf tubes and stored in ice. For the assay, 20 μL of this supernatant and 100 μL of luciferase assay substrate (Promega) were used. The lysate and the substrate were both thawed to RT before performing the assay. The substrate was added to the lysate, and the luciferase activity was measured in a luminometer (Turner designs, 20/20, Promega, Madison, WI, USA) in standard single-luminescence mode. The integration time of measurement was 10,000 ms and a delay of 2 s was given before each measurement. The protein concentration in the cell lysate supernatant was estimated in each case with Lowry protein assay kit (PIERCE, Rockford, IL, USA). Comparison of the transfection efficiencies of the individual bolas was made based on the data for luciferase expressed as relative light units (RLU)/mg of protein. All the experiments were done in triplicate, and the results presented are the average of at least two such independent experiments done on the same days.

### 2.6. Cytotoxicity Assays

Toxicities of bolaplexes toward HEK 293 cells, HeLa cells, HepG-2 cells, and A549 cells were determined by using MTS reduction assay following literature procedures. About 1.0 × 10^4^ cells/well were seeded into 96-well plates. After 24 h, optimized bola/DOPE formulations were completed with 0.2 μg of pEGFP-N1 DNA at various N/P ratios for 30 min; 100 μL of bolaplexes were added to the cells in the absence of serum. After 4 h of incubation, bolaplexes solutions were removed, and 100 μL of media with 10% FBS was added. After 24 h, 20 μL MTS and 80 μL PBS were added to each well and the plates were incubated at 37 °C for another 0.5 h. Then, the absorbance of each sample was measured using an ELISA plate reader (model 680, BioRad, Hercules, CA, USA) at a wavelength of 490 nm. The cell viability (%) was obtained according to the manufacturer’s instruction. Lipoplex prepared from Lipofectamine 2000 was used as control.

### 2.7. Flow Cytometry Assay

The cellular uptake of the bolasome/fluorescein labelled-DNA complexes was analyzed by flow cytometry. The Label IT Cy5 Labeling Kit was used to label pGL-3 with Cy5 according to the manufacturer’s protocol. Briefly, HEK 293 cells and HeLa cells were seeded into 24-well plates (1.0 × 10^5^ cells/well) and allowed to attach and grow for 24 h. Before transfection, the medium was replaced with serum-free culture medium. Cells were incubated with Cy5 labelled DNA nanoparticles (0.8 μg DNA/well, optimal N/P ratio of each sample) in media for 4 h at 37 °C. Subsequently, the cells were washed with 1 × PBS and 155 U/mL of heparin sodium aqueous solution and harvested with 0.25% Trypsin/EDTA and resuspended in 1 × PBS. Cy5-labelled plasmid DNA uptake was measured in the FL4 channel using the red diode laser (633 nm). Data from 10,000 events were gated using forward and side scatter parameters to exclude cell debris. The flow cytometer (BD Accuri^TM^ C6) was calibrated for each run to obtain a background level of ~1% for control samples (i.e., untreated cells).

### 2.8. Confocal Laser Scanning Microscopy (CLSM)

HeLa cells were seeded at a density of 2.5 × 10^5^ cells per well in 35 mm confocal dish (Φ = 15 mm), 24 h prior to transfection. For transfection in the absence of serum, the medium was exchanged with serum-free medium (for transfection with serum, the medium was exchanged with serum-containing medium). Complexes of bolasomes and Cy5-labelled pGL-3 at a given concentration were added to each well. After incubation at 37 °C for 4 h, cells were rinsed 3 times with PBS (pH 7.4), fixed with 500 μL 4% paraformaldehyde (dissolved with PBS buffer) for 15 min, nuclear staining was done with Hoechst 33342. The CLSM observation was performed using confocal laser scanning microscope (CLSM, ZEISS LSM 780) at excitation wavelengths of 405 nm for Hoechst 33342 (blue), 633 nm for Cy5 (red), respectively. For the endosome escape experiment, after incubation at 37 °C for different times (4 h, 8 h, 12 h, and 18 h), LysoTracker green (1:750) was added into the well for another 30 min incubation at 37 °C for lysosome staining. Then cells were rinsed 3 times with PBS (pH 7.4), fixed with 500 μL 4% paraformaldehyde (dissolved with PBS buffer) for 15 min and nuclear staining was done with Hoechst 33342 for 10 min. The CLSM observation was performed using confocal laser scanning microscope (CLSM, ZEISS LSM 780) at excitation wavelengths of 504 nm for LysoTracker green (green), 633 nm for Cy5 (red), respectively.

For DOX internalization assay, cells were incubated with DOX-loaded bolaamphiphile aggregates (0.5 μg/mL DOX) and DOX·HCl (0.5 μg/mL DOX) in media for 8, 12, and 24 h a t 37 °C. Finally, cells were rinsed twice with PBS (pH 7.4) and fixed with 4% paraformaldehyde for 10 min. The CLSM observation was performed using ZEISS LSM 780 at excitation wavelengths of 405 nm for Hoechst 33342 (blue), 488 nm for DOX (red), respectively.

### 2.9. Determination of the Critical Aggregation Concentration (CAC)

The critical aggregation concentration was determined using pyrene as a fluorescence probe. The bolaamphiphile concentration varied from 1.0 × 10^−6^ mg mL^−1^ to 1 mg mL^−1^, and the pyrene concentration was fixed at 6.0 × 10^−7^ M. The fluorescence spectra were recorded using a HITACHI F-7000 Fluorescence Spectrophotometer (Hitachi High-Tech Science, Schaumburg, IL, USA). Both the emission and excitation slit widths were 5 nm. The samples were excited at 335 nm and the emission spectra were recorded from 350 to 500 nm. The emission fluorescence values, I_373_ and I_383_ at 373 nm and 383 nm, respectively, were used for the subsequent calculations. The CAC was determined from the plots of the I_383_/I_373_ ratio versus the logarithm of the polymer concentration using the intersection of the linear regression lines as the CAC value.

### 2.10. Loading of DOX into Bolaamphiphiles Aggregates

Hydrophobic DOX was prepared according to a previous report [34]. Then the hydrophobic DOX (1.0 mg) and bolaamphiphiles (5.0 mg) were dissolved in anhydrous CHCl_3_ (5.0 mL) in an autoclaved glass vial. The solvent was then slowly rotary-evaporated at room temperature. The last trace of organic solvent was removed by keeping these films under vacuum above 12 h. Deionized water (5.0 mL) was added to the autoclaved glass vial and the mixture was vortexed vigorously until the materials were completely resuspended. Sonication of these suspensions with an ultrasonic probe (200 w) for 5 min in an ice bath was carried out and then the mixed solution was stirred for 24 h at room temperature (RT). Then the mixed solution was filtered with a 450 nm film filter to remove the unloaded DOX and finally obtain the DOX-loaded bolaamphiphiles.

The encapsulation efficiency (EE) was calculated using the following formula:

*EE* = W/W_0_ × 100%,

where W was the weight of DOX in the aggregates after filtration. W_0_ was the weight of DOX initially added in the aggregates preparation.

The drug-loading capacity (DL) was calculated using the following equation:

*DL* = W_drug_/W_total_ × 100%,

where W_drug_ is the weight of drug and W_total_ is the total weight of drug and bolaamphiphiles.

## 3. Results and Discussion

### 3.1. Synthesis of the Bolaamphiphiles

A series of novel asymmetrical cationic bolaamphiphiles containing a protonated lysine (Lys) headgroup and a functional hydrophilic groups on the other side of the molecule were designed and synthesized. The functional groups include pH-sensitive histidine (His) or a targeting moiety such as triphenylphosphonium (TPP), biotin or galactose (Gal). Lysine has excellent DNA binding ability due to its two primary amines, therefore it has been widely used as a hydrophilic head in various biomaterials. Histidine contains an imidazole group, whose amine group has a suitable p*K*a close to the more acidic endosome environment (pH 5.0–6.5), thus it was hoped to afford the material better pH buffering capacity and more efficient endosome escape. TPP might achieve mitochondria-targeting delivery [30], while biotin and galactose could improve cellular uptake of biomaterials through ligand-receptor recognitions in certain cells. As shown in Scheme 1, the precursor compound **1** with hydrophobic skeleton, which was obtained according to our previous report [26], was first protected by *tert*-butyldimethylsilyl chloride (TBS-Cl) to give the mono-protected compound **2**. Then the free hydroxyl was reacted with 2Boc-lysine by esterification in the presence of dicyclohexylcarbodiimide (DCC) and 4-dimethylaminopyridine (DMAP). Subsequent TBS deprotection by tetrabutylammonium fluoride (TBAF) yielded compound **3** (**2Boc-Lys-14-10-OH**). The four target bolaamphiphiles was obtained by the condensation between **3** and respective acids **A1**–**A4**, and subsequent deprotection of boc groups. These bola-lipids were termed using their hydrophilic moieties, namely **Lys-His**, **Lys-TPP**, **Lys-Biotin** and **Lys-Gal**. For comparison study, we also used a symmetric bolaamphiphile **Lys-14-10**, which has an optimized chain length from our previous studies, as counterpart. All novel compounds in each step were purified and their structures were confirmed by ^1^H NMR, ^13^C NMR, and HRMS.

### 3.2. Interaction between the Bolasomes and DNA

**Figure 1:**
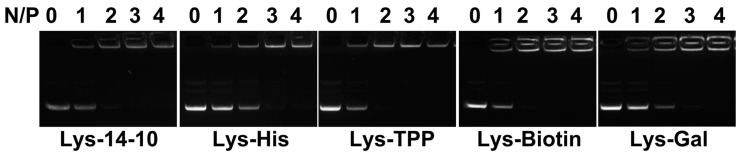
Electrophoretic gel retardation assays of bolasome/pDNA complexes at different N/P ratios. The molar ratio of bolaamphiphile/DOPE was 1:1.

Cationic lipids for gene delivery have been frequently combined with neutral lipids such as DOPE, which might increase the TE significantly due to its special membrane fusion ability [35]. The transfection potential was substantially improved when **Lys-14-10** was mixed with an equimolar amount of DOPE in our previous work [26]. Therefore, the bolaamphiphiles/DOPE ratio of 1:1 was used to prepare bolasomes by the thin film hydration method herein. The agarose-gel retardation assay was first employed to evaluate their plasmid DNA (pUC 19) binding abilities. Results in Figure 1 show that complete DNA retardation could be achieved at an N/P of 4 for the bolasomes formed from all five bolaamphiphiles, indicating their good DNA binding ability. **Lys-His** and **Lys-Gal** showed a slightly lower binding ability. The ethidium bromide (EB) exclusion assay was also carried out to further evaluate the binding ability of the bolasomes. EB is highly fluorescent when intercalated into DNA base pairs. The addition of another DNA binding reagent with higher affinity may displace EB from its intercalation site, leading to fluorescence quenching. Most of the materials can significantly decrease the fluorescent intensity along with an increase of N/P ratio over 0–2 (Figure S1), suggesting that the EB was displaced by the bolasomes. Similar to the gel retardation results, **Lys-Gal** and **Lys-His**, especially the former, showed lower quenching ability. This might be attributed to the oxygen-rich structure of galactose, which may screen the positive charge of liposome, resulting in weaker DNA binding. The decrease of fluorescence intensity became much slighter with further increase of N/P ratio (>3), indicating full DNA condensation [36].

**Figure 2:**
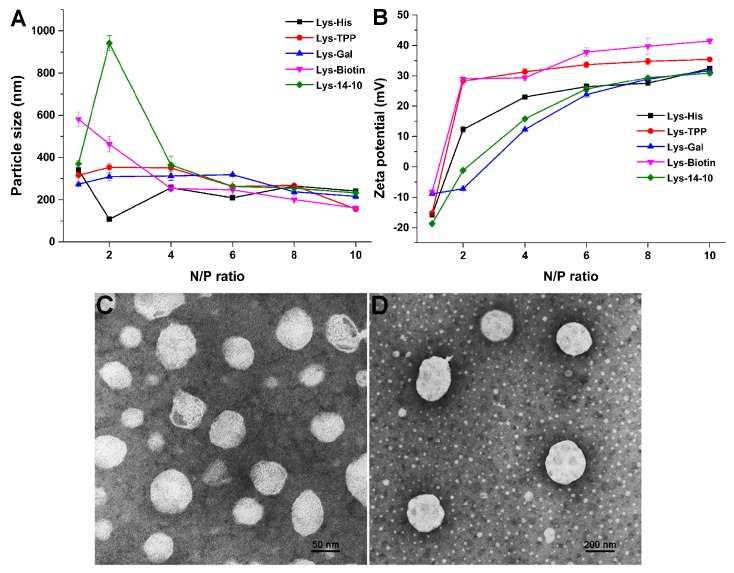
Characterization of the bolaplexes. (**A**) Mean particle sizes; (**B**) zeta-potentials. Data represent mean ± SD (*n* = 3). (**C**,**D**) TEM images of bolasome and bolaplex (N/P = 4) formed from **Lys-His**.

The physicochemical characteristics of biomaterials may tremendously influence their subsequent transfection performances. The average particle size, surface charge, and morphology of the self-assembly particles were investigated by using dynamic light scattering (DLS) method and transmission electronic microscope (TEM). The bolasomes, before binding to DNA, were first studied to estimate the self-assembly of the bolaamphiphiles. Compared to the symmetric **Lys-14-10**, the asymmetric amphiphiles containing TPP and Gal could form bolasomes with similar size of ~85 nm in diameter. **Lys-Biotin** formed larger particles (115 nm in diameter), while **Lys-His** could form much smaller bolasome with average diameter of 65 nm (Figure S2). It was reported that such size range is suitable for DNA binding and delivery [37], and smaller particle size might receive easier endocytosis [38]. The functional moiety seemed not to affect the surface potential of the bolasomes, and the zeta potentials of all bolasomes were found to be similar around +45 mV (Figure S2). Subsequently, these parameters of the bolasomes/DNA complexes (bolaplexes) were also measured, and the results are shown in Figure 2. After binding with pUC 19 plasmid DNA to form bolaplexes, the particle sizes observably increased. For the incomplete DNA condensation under low bolasome dosage, the particle sizes were disordered at low N/P ratio range. From the N/P ratio of 4, the diameters of the bolaplexes became stable at 250–300 nm, indicating a full DNA condensation. Surface potentials of the bolaplexes gradually rose along with the increase of the N/P ratio, and finally reached +25–35 mV above an N/P of 6 (Figure 2B). Compared to symmetric **Lys-14-10**, the surface potentials of the bolaplexes formed from asymmetric ones were higher except **Lys-Gal**, whose oxygen-rich structure on galactose might have screened the positive charges. Such results were consistent with those obtained in gel electrophoresis and EB exclusion assay. Further, transmission electron microscopy (TEM) images revealed that the bolasomes formed from **Lys-His** gave spherical nanoparticles with diameters about 50 nm (Figure 2C). The bolaplexes also had spherical shape, while the size was bigger (~200 nm) than the empty bolasomes (Figure 2D).

### 3.3. In Vitro Gene Transfection Studies

**Figure 3:**
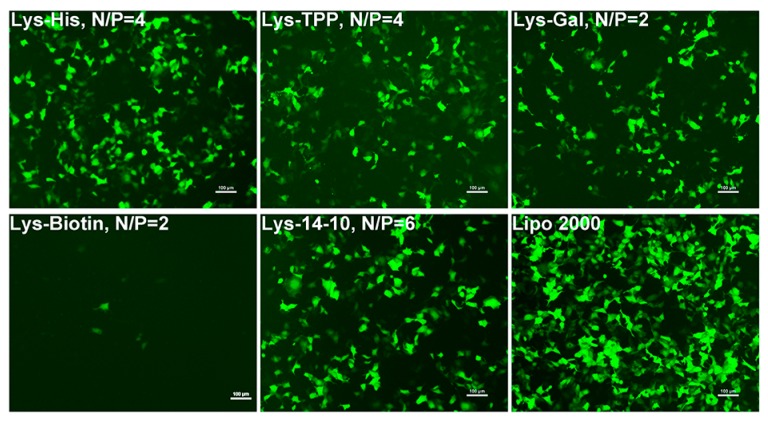
Fluorescent microscopy images of HEK 293 cells transfected by bolaplexes at the optimal N/P ratio (with the vector name and N/P ratio in each image). The molar ratio of bolaamphiphile/DOPE was 1:1, lipofectamine 2000 was used for comparison. The cells were observed by fluorescence microscopy after a 24 h transfection. Scale bar: 100 μm.

**Figure 4:**
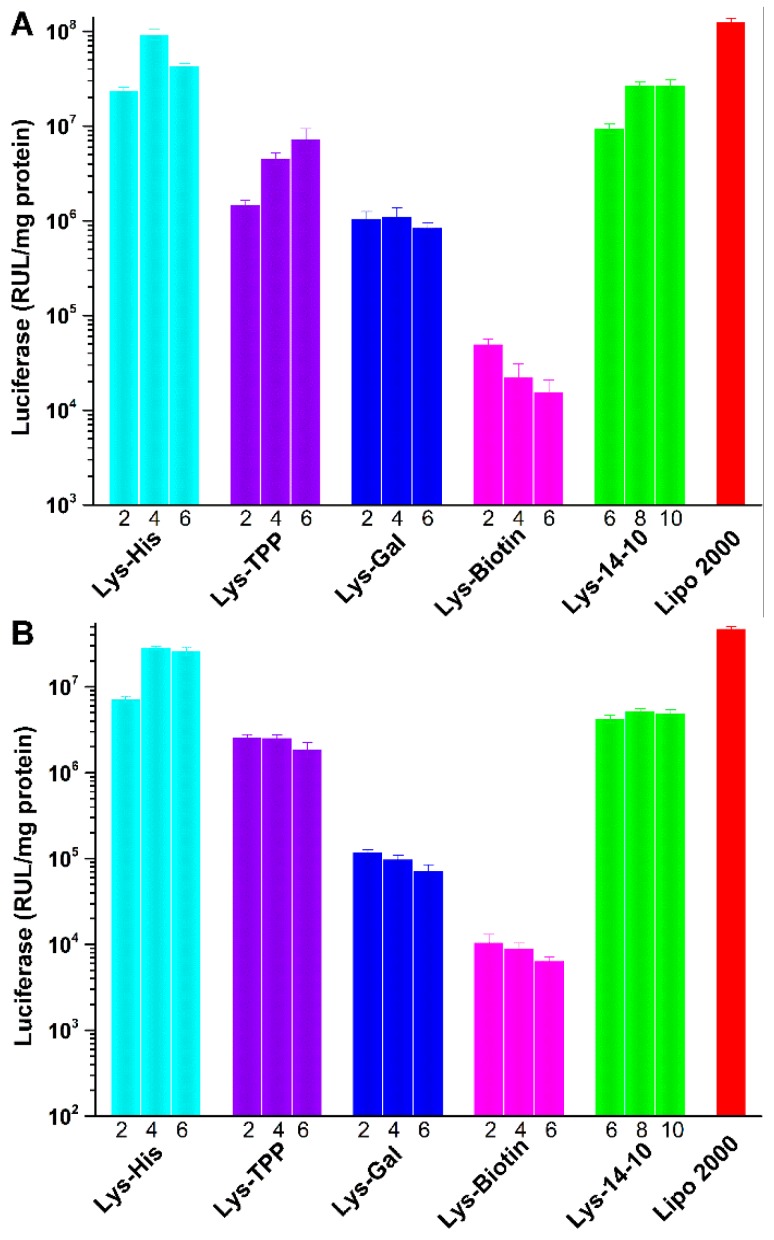
Luciferase expression in HEK 293 (**A**) and HeLa (**B**) cells transfected by bolaplexes at various N/P ratios. Data represent mean ± SD (*n* = 3).

To evaluate the potential of the asymmetric bolaamphiphiles as gene delivery vector, in vitro green fluorescent protein (GFP) expression experiment was first studied in HEK 293 cells. Figure 3 shows the eGFP expression mediated by the bolasomes in HEK 293 cells at optimal N/P ratio observed using an inverted fluorescence microscope. The density of the green fluorescence induced by **Lys-His**, which has a pH-sensitive imidazole group, was higher than that in the cells transfected by other bolaamphiphiles, including the symmetric one **Lys-14-10**. The bolasomes containing targeted moiety (**Lys-TPP**, **Lys-Gal** or **Lys-Biotin**) only gave moderate eGFP expression, and **Lys-Biotin** induced a poor green fluorescence signal, which might be due to the serious precipitation during the incubation with eGFP reporter gene. Subsequently, luciferase experiment was used to quantitatively study the TE of these bolaplexes in HEK 293 and HeLa cells. As shown in Figure 4, the results were consistent with those obtained in the GFP assays. **Lys-His** gave the best TE in both cell lines, and its TE was also higher than the symmetric counterpart **Lys-14-10** and comparable to lipofectamine 2000. We consider that the imidazole in the histidine moiety might play a crucial role for the improvement of the TE compared to **Lys-14-10**. HeLa cell line was chosen for the fact that it was one of the reported biotin targeted tumor cell lines [39]. However, unfortunately, due to the same reason in eGFP assay (precipitation), **Lys-Biotin** did not exhibit good transfection result toward HeLa cells. Similarly, with hepatoma cells targeting group galactose in the structure, **Lys-Gal** could not achieve the expected transfection results toward HepG2 cells (data not shown).

**Figure 5:**
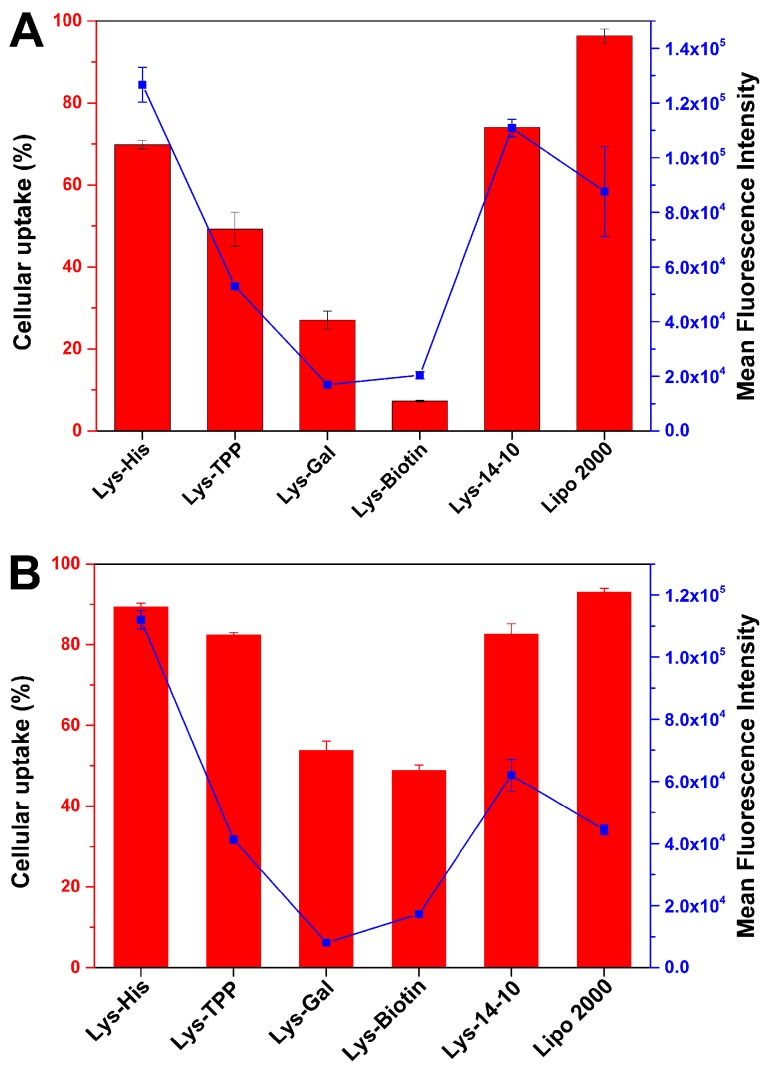
The uptake cell percentage (columns, Cy5-positive cells) and mean fluorescence intensity (dots and lines) of bolaplexes at the optimal N/P ratio in HEK 293 (**A**) and HeLa (**B**) cells quantified by flow cytometry. Lipofectamine 2000 was used as the control. Data represent mean ± SD (*n* = 3).

To figure out the transfection mechanism mediated by these bolasomes, and also the reason for the superiority of **Lys-His** for the gene transfection, several assays were performed to elucidate the fate of the cargo genes. Cellular uptake of the vector/DNA complexes was estimated through fluorescence-activated cell sorting (FACS) technique. After 4 h cell incubation with the complexes at each optimized N/P ratio in HEK 293 cells, the percentage of Cy5-positive cells and the mean fluorescence intensity (MFI) of Cy5 were calculated and are shown in Figure 5A. It was found that **Lys-His** had a much higher cellular uptake (both uptake cell percentage and MFI) than other asymmetric bolaamphiphiles, and the percentage (~70%) of Cy5-positive cells was similar to that of the symmetric **Lys-14-10**. Although such positive cell percentage was lower than that induced by lipofectamine 2000, the MFI was much higher, indicating a higher amount of labeled DNA was internalized into the cells. On the other hand, **Lys-TPP**, **Lys-Gal** and **Lys-Biotin** gave much lower cellular uptake, which might be the main reason for their lower TE. **Lys-His** could give better performance in the same assay in HeLa cells, in which comparable positive cell percentage and much higher MFI compared to lipofectamine 2000 were obtained (Figure 5B). Such results could also be visually verified by confocal laser scanning microscopy (CLSM, Figure S3). After 4 h of incubation with the complexes, **Lys-His** and **Lys-14-10** induced comparable red fluorescent signals, which were slightly stronger than that by lipofectamine 2000. From these results, it was believed that **Lys-His** might mediate the internalization of DNA cargo with high efficiency. After that, the uptake mechanism of **Lys-His**/DNA bolaplex was further studied. Transfection was performed in HeLa cells in the presence of chemical uptake inhibitors including cytochalasin D, genistein, chlorpromazine, and nocodazole, which may inhibit macropinocytosis, caveolae-pathway, clathrin-pathway, and microtubule-mediated endocytosis, respectively. Results in Figure S4 reveal that genistein and chlorpromazine did not inhibit the uptake at all. On the contrary, nocodazole severely hindered the uptake, and an approximated 55% reduction of positive cells was observed, suggesting that the internalization of the bolaplex is mainly microtubule-mediated endocytosis. Moreover, cytochalasin D also resulted in an about 20% inhibition of cellular uptake, indicating that macropinocytosis might take place synergically. 

**Figure 6:**
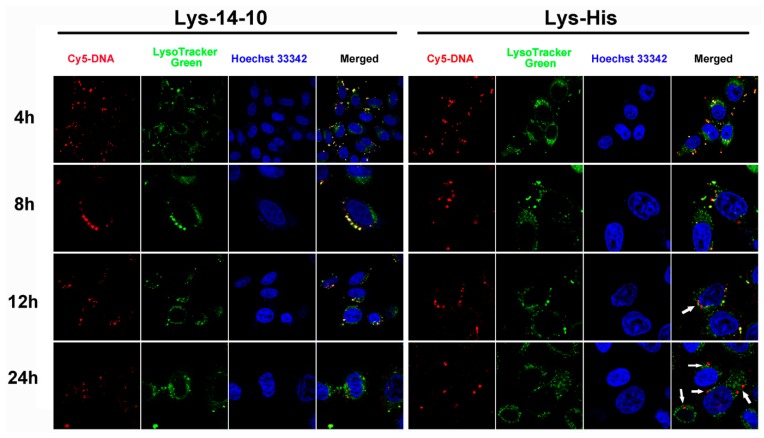
Confocal laser scanning microscopy (CLSM) images of HeLa cells transfected with Cy5-labelled DNA by **Lys-14-10** and **Lys-His** at the optimal transfection N/P ratio at different times. For each row, from left to right: Cy5-labelled pGL-3 DNA (red); lysosomes and acidic late endosomes stained by LysoTracker green (green); cell nucleus stained by Hoechst 33342 (blue) and merged image.

The imidazole group in **Lys-His** with p*K*a of ~6 may contribute to its endosomal pH buffering capacity and subsequent transfection performances. Acid-base titration experiment results confirmed the good buffering capacity of **Lys-His**, which was better than the symmetric counterpart **Lys-14-10** and also 25 kDa PEI, especially in the endosome pH range (Figure S5). Such buffering capacity would afford the material “proton-sponge effect” and facilitate the endosome escape. To further prove this, CLSM was applied to compare the endosomal escape behaviors of the bolasomes formed from **Lys-His** and **Lys-14-10** in HeLa cells. DNA cargo, lysosome and cell nucleus were labeled/stained with Cy-5, LysoTracker green and Hoechst 33342, respectively. The images were taken after 4, 8, 12, and 24 h of transfection (Figure 6). It was shown that after 4 or 8 h, abundant yellow colocalization fluorescent signals were found in both bolasome-mediated transfection, indicating that most internalized DNA or bolaplexes was trapped in late endosomes/lysosomes. With the extension of incubation time, the yellow dots markedly decreased and red signals appeared in **Lys-His** mediated transfection, especially after 24 h transfection, almost all red fluorescent signals were completely separated from green fluorescent signals (white arrows indicated), suggesting the effective escape of DNA or **Lys-His**/DNA bolaplex from the endosome. For comparison, in the transfection with **Lys-14-10**, although a few red signals were found, there were still many yellow colocalization signals after 12 or 24 h transfection. Such results demonstrate that bolaplex formed from imidazole-contained **Lys-His** has better endosomal escape ability, which may contribute to its higher TE.

### 3.4. Cytotoxicity

**Figure 7:**
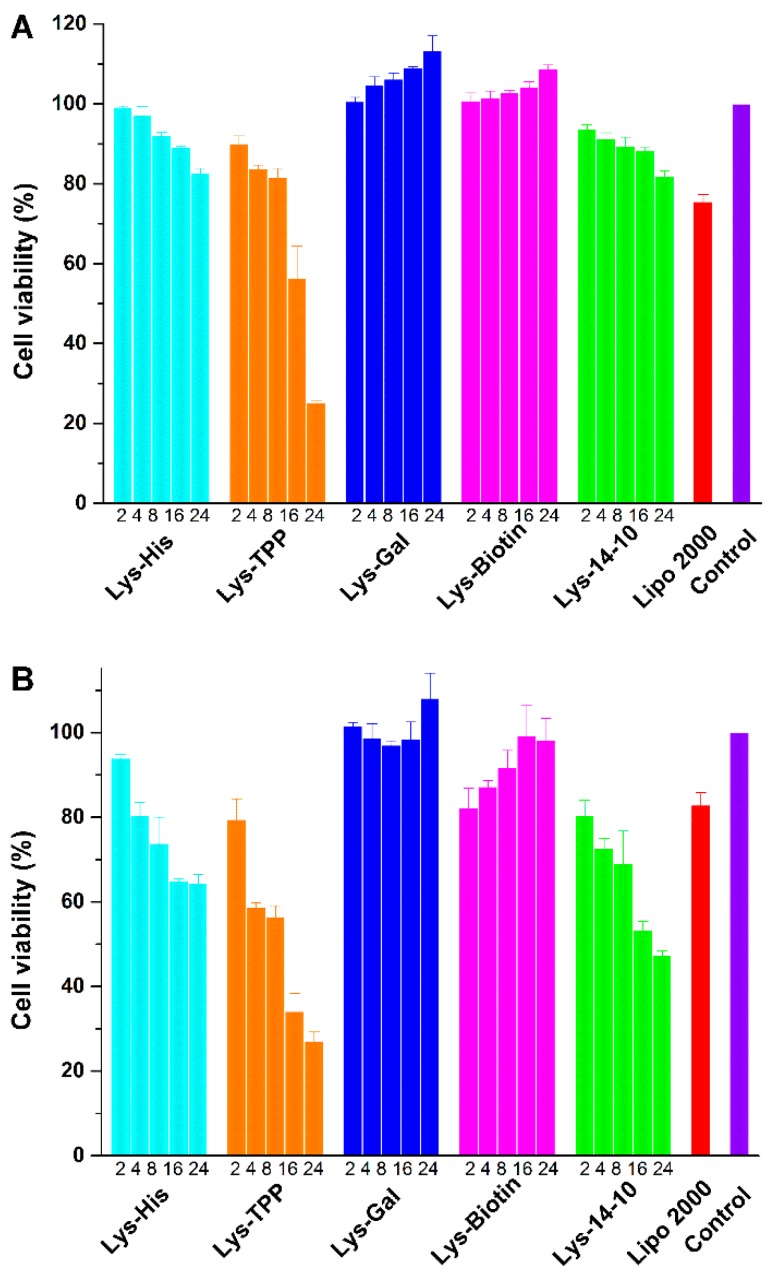
In vitro cytotoxicity of the bolaplexes at various N/P ratios in HEK 293 (**A**) and HeLa cells (**B**) for a 24 h incubation. Data represent mean ± SD (*n* = 3).

Low cytotoxicity is essential for a synthetic gene vector in clinical applications. The cytotoxicities of the prepared bolaplexes at various N/P ratios were estimated in various cell lines by MTS-based assay. The commercial transfection reagent Lipofectamine 2000 was used for comparison. As shown in Figure 7A, the bolaplexes did not induce significant cytotoxicity against HEK 293 cells, and the cell viability percentages were higher than 90% at transfection N/P ratios (<16) except **Lys-TPP**. The cytotoxicity of the bolaamphiphiles, especially at transfection N/P ratio, was lower than Lipofectamine 2000, and similar results were found in HepG2 and A549 cell lines (Figure S6). Although these materials induced more cell death in HeLa cell line (Figure 7B), the cytotoxicity at relatively low N/P was acceptable. Compared to **Lys 14-10**, the asymmetric **Lys-His** resulted in similar cell viability in each cell line, indicating that the introduction of the imidazole group would not bring excess toxicity. It is worth noting that the bolasomes formed from **Lys-Gal** and **Lys-Biotin** slightly precipitated during the incubation with plasmid DNA in opti-MEM culture medium under high N/P ratios (>8), resulting in lower cellular uptake and almost no toxicity.

### 3.5. In Vitro Drug Delivery Studies

The bolaamphiphilic vesicle has also been demonstrated as a promising drug delivery system [40]. These bolaamphiphiles studied herein could self-assemble into spherical aggregates with 20–40 nm diameters (Figure S7) with very low critical aggregation concentration (CAC), which was measured by fluorescence spectrometry using a pyrene probe (Figure S8). Results revealed that the CACs of functionalized asymmetric bolaamphiphiles were 0.015–0.031 mg/mL, which were significantly lower than that of symmetric **Lys-14-10** (0.061 mg/mL, Table 1). Doxorubicin (DOX) was then used to estimate the encapsulation ability of the bolasomes, which were prepared via similar methods without the use of DOPE. The DOX-loaded nanoparticles (represented as DOX@**Lys-X**, **X** means the other functional group) were prepared though hydrophobic interaction and formed a clear and transparent solution, which could be stable for several months at 4 °C storage without any precipitates (Figure S9). After encapsulating DOX, the particle sizes (z-average) of drug-loaded nanoparticles increased obviously with decreased polydispersity index (PDI) value, while the zeta-potentials were almost unchanged (Figure S10 and Table 1). The loading of a hydrophobic DOX resulted in a satisfactory encapsulation efficiency (EE, average 70.5–97.5%) and drug-loading capacity (DL, average 12.4–16.3 wt %, Table 1). To our delight, **Lys-His**, which has the best gene transfection performance, also exhibited the highest EE (97.5% ± 0.5%) and DL (16.3% ± 0.07%, theoretical DL: 16.7%), suggesting its excellent capability for drug loading.

**Figure 8:**
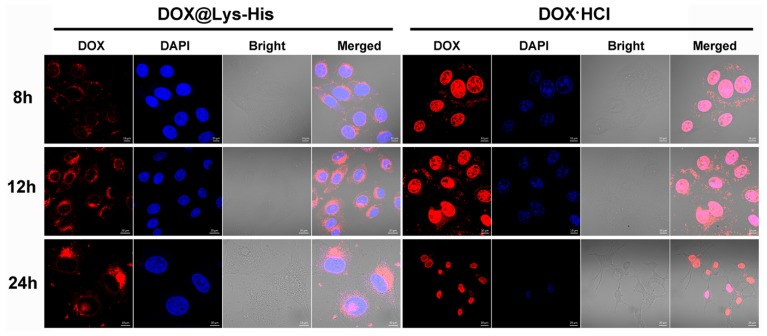
Confocal laser scanning microscopy (CLSM) of HepG-2 cells treated with DOX@**Lys-His** and free DOX·HCl with [DOX] = 0.5 μg/mL at different times. For each row, from left to right: DOX (red); cell nucleus stained by 4′,6-diamidino-2-phenylindole (DAPI) (blue); bright field and merged image.

**Figure 9:**
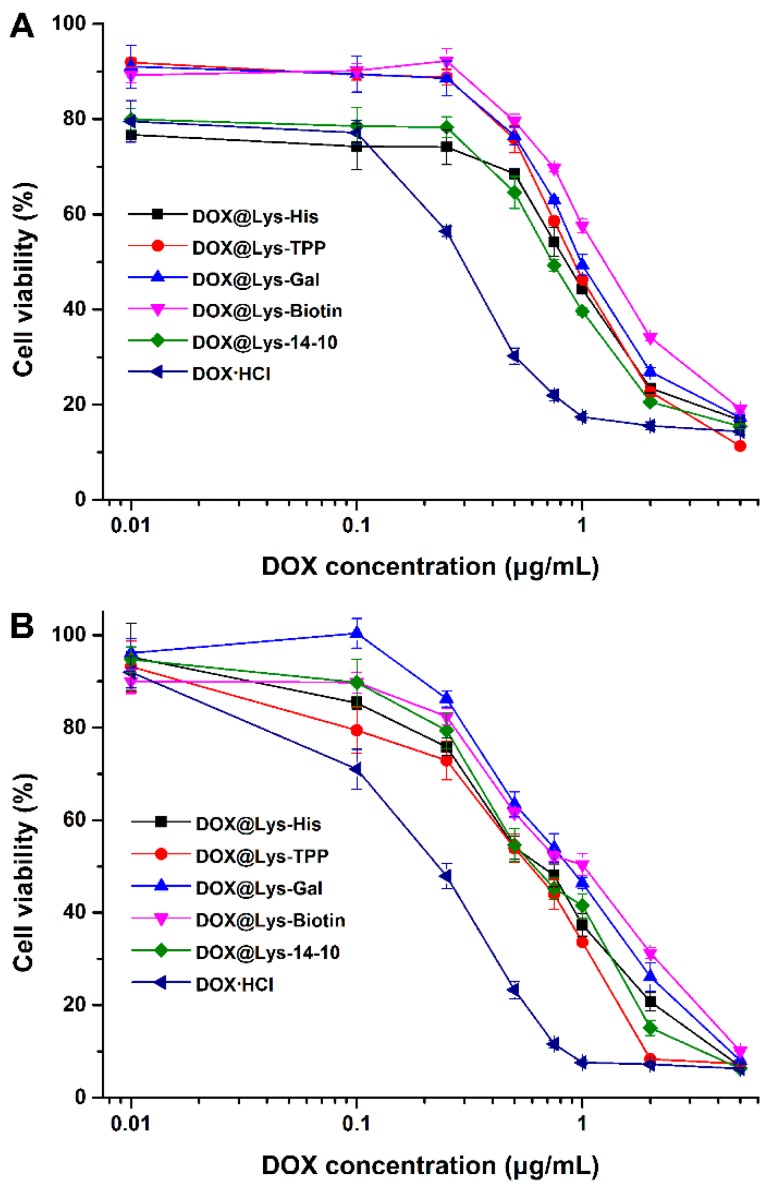
In vitro antitumor effects of DOX-loaded bolaamphiphiles nanoparticles in HepG-2 (**A**) and HeLa (**B**) cells treated with various concentrations DOX-loaded NPs for 48 h. Data represent mean ± SD (*n* = 3).

Subsequently, the intracellular distribution of DOX@**Lys-His** NPs was monitored by confocal laser scanning microscopy. Both the intracellular intensity and location of the DOX fluorescence could strongly influence the antitumor effects of the drug. As shown in Figure 8, the red fluorescence corresponds to the intracellular DOX, while the blue fluorescence indicates nucleus stained by DAPI. After 8 h incubation, for DOX@**Lys-His** group, only a small quantity of red fluorescence signals was found around the nucleus, while the free DOX·HCl group had already been localized in the nucleus at this time. However, with the extension of incubation time, an increasing amount of the red fluorescent signals accumulated in the perinuclear region, and started to enter the nuclei after 24 h incubation. For comparison, the nuclei were damaged and shrunk by free DOX·HCl after 24 h. Such results indicated the successful delivery and controlled release of DOX to the nucleus by **Lys-His**-based nano-vector. The cell viability assay results revealed that the drug-free bolaamphiphiles exhibited very low toxicity toward tumor cells (HepG-2 and HeLa cells) except **Lys-TPP** under relatively high concentration (Figure S11). However, after treating with DOX-loaded bolasomes for 48 h, the cell viability was severely lowered, and the IC_50_ values of these DOX-loaded NPs were approximately 0.5–1.0 µg mL^−1^ (Figure 9), suggesting good in vitro antitumor effect.

## 4. Conclusions

In summary, a series of asymmetric bolaamphiphiles containing different functional groups were designed and synthesized. The potentials of these bolaamphiphile-based nanoparticles as versatile vectors for both nucleic acids and chemical drugs were studied. Results revealed that these bola-type molecules are all capable of forming bolasomes with good DNA binding and condensation ability. The asymmetric bolaamphiphiles could give higher gene transfection efficiency than symmetric counterparts and comparable efficiency to commercially available transfection reagent. Mechanism studies confirmed that the better transfection efficiency of **Lys-His** came from its high cellular uptake and subsequent good endosomal escape, which was attributed to the pH buffering capacity of the imidazole moiety. On the other side, these bolaamphiphiles exhibited good drug loading ability, and they could encapsulate the hydrophobic drug doxorubicin with a high drug loading content and encapsulation efficiency. CLSM experiment and cell viability assay exhibited a controlled release of the drug with the assistance of bolasomes. It was shown that such bolaamphiphiles have great potential as nano-vectors for both DNA and drug delivery. Moreover, it is logical to apply such materials for gene/drug co-delivery, and the relevant studies are now in progress.

## Figures and Tables

**Table 1:** Characterization of drug-free and drug-loaded bolaamphiphiles nanoparticles (NPs).

Material	NP Preparation			CAC [mg/mL]	Drug Loading	
	Size [nm]	ζ-Potential [mV]	PDI		EE [%]	DL [%]
**Lys-His**	119.9 ± 3.1	37.7 ± 4.0	0.311 ± 0.072	0.022	-	-
**Lys-TPP**	122.0 ± 2.2	34.5 ± 1.7	0.258 ± 0.017	0.015	-	-
**Lys-Gal**	91.4 ± 5.3	28.7 ± 1.6	0.357 ± 0.038	0.021	-	-
**Lys-Biotin**	105.0 ± 1.4	33.5 ± 1.3	0.316 ± 0.020	0.031	-	-
**Lys-14-10**	109.7 ± 0.4	42.2 ± 4.3	0.214 ± 0.020	0.061	-	-
DOX@**Lys-His**	149.2 ± 1.7	36.6 ± 1.6	0.219 ± 0.003	-	97.5 ± 0.5	16.3 ± 0.07
DOX@**Lys-TPP**	158.0 ± 2.4	32.8 ± 0.9	0.119 ± 0.017	-	76.3 ± 0.3	13.2 ± 0.04
DOX@**Lys-Gal**	131.7 ± 3.1	34.9 ± 0.6	0.318 ± 0.026	-	70.5 ± 0.5	12.4 ± 0.08
DOX@**Lys-Biotin**	123.1 ± 0.4	41.3 ± 1.1	0.211 ± 0.012	-	86.5 ± 0.5	14.7 ± 0.07
DOX@**Lys-14-10**	198.1 ± 2.5	39.0 ± 0.6	0.154 ± 0.007	-	81.0 ± 0.5	13.9 ± 0.07

## Supplementary Materials

The following are available online at http://www.mdpi.com/2079-4991/8/2/115/s1, Figure S1: Fluorescent quenching assay of EB/DNA with the addition of bolasomes. The molar ratio of bolaamphiphile/DOPE was 1:1, Figure S2: Mean particle sizes (columns) and zeta potentials (dots) of the five bolasomes with the DOPE/bolaamphiphile ratio of 1:1 obtained by DLS at room temperature. Data represent mean ± SD (*n* = 3), Figure S3: CLSM images of HeLa cells transfected with Cy5-labelled DNA by the bolaplexes at the optimal transfection N/P ratio. For each row, left: cell nuclei stained by Hoechst 33342 (blue); middle: Cy5-labelled pGL-3 DNA (red); right: merged image. Scale bar: 10 μm, Figure S4: Relative cellular uptake of **Lys-His**/DNA bolaplexes at optimal transfection N/P ratio in HeLa cells in the presence of various endocytic inhibitors quantified by flow cytometry analysis, Figure S5: Acid-base titration profiles of **Lys-14-10**, **Lys-His**, 25 kDa PEI and 150 mM NaCl solutions. Bolaamphiphiles or PEI (0.050 mmol of amino groups) was first treated with 1 N HCl to adjust pH to 2.0, and then the solution pH was measured after each addition of 20 μL of 0.1 N NaOH, Figure S6: In vitro cytotoxicity of the bolaplexes at various N/P ratios in HepG-2 (A) and A549 cells (B) for a 24 h incubation. Data represent mean ± SD (*n* = 3), Figure S7: TEM images of the bolaamphiphile aggregates without DOPE, Figure S8: Plots of the intensity ratio I_383_/I_373_ from the pyrene emission spectra versus the logarithm of the concentration for self-assembling aggregates in aqueous media from **Lys-His**, **Lys-TPP**, **Lys-Gal,** and **Lys-Biotin**, respectively, Figure S9: Aqueous drug-loaded bolaamphiphile solutions after storage under 4 °C for 5 months, Figure S10: Mean particle sizes (A) and zeta-potentials (B) of drug-free bolaamphiphiles nanoparticles. Mean particle sizes (C) and zeta-potentials (D) of drug-loaded bolaamphiphile nanoparticles measured by DLS at room temperature at a fixed angle (90°). Data represent mean ± SD (*n* = 3), Figure S11: In vitro cytotoxicity of DOX-free bolaamphiphile nanoparticles in HepG-2 (A) and HeLa (B) cells treated with various concentrations blank materials for 48 h. Data represent mean ± SD (*n* = 3).

## References

[1] Verma I.M., Somia N. (1997). Gene therapy—Promises, problems and prospects. Nature.

[2] Bhattacharya S., Bajaj A. (2009). Advances in gene delivery through molecular design of cationic lipids. Chem. Commun..

[3] Mintzer M.A., Simanek E.E. (2009). Nonviral vectors for gene delivery. Chem. Rev..

[4] Guo X., Huang L. (2012). Recent advances in nonviral vectors for gene delivery. Acc. Chem. Res..

[5] Smith A.E., Helenius A. (2004). How viruses enter animal cells. Science.

[6] Pouton C.W., Seymour L.W. (2001). Key issues in non-viral gene delivery. Adv. Drug Deliv. Rev..

[7] Liu Q., Jiang Q.-Q., Yi W.-J., Zhang J., Zhang X.-C., Wu M.-B., Zhang Y.-M., Zhu W., Yu X.-Q. (2013). Novel imidazole-functionalized cyclen cationic lipids: Synthesis and application as non-viral gene vectors. Bioorg. Med. Chem..

[8] Zhang Y.-M., Liu Y.-H., Zhang J., Liu Q., Huang Z., Yu X.-Q. (2014). Cationic gemini lipids with cyclen headgroups: Interaction with DNA and gene delivery abilities. RSC Adv..

[9] Huang Z., Liu Y.-H., Zhang Y.-M., Zhang J., Liu Q., Yu X.-Q. (2015). Cyclen-based cationic lipids containing a ph-sensitive moiety as gene delivery vectors. Org. Biomol. Chem..

[10] Zhang Q.-F., Yi W.-J., Wang B., Zhang J., Ren L., Chen Q.-M., Guo L., Yu X.-Q. (2013). Linear polycations by ring-opening polymerization as non-viral gene delivery vectors. Biomaterials.

[11] Yi W.-J., Yu X.-C., Wang B., Zhang J., Yu Q.-Y., Zhou X.-D., Yu X.-Q. (2014). Tacn-based oligomers with aromatic backbones for efficient nucleic acid delivery. Chem. Commun..

[12] Guo Q., Liu Y.-H., Xun M.-M., Zhang J., Huang Z., Zhou X.-D., Yu X.-Q. (2015). Diol glycidyl ether-bridged low molecular weight PEI as potential gene delivery vehicles. J. Mater. Chem. B.

[13] Liu H., Wang Y., Wang M., Xiao J., Cheng Y. (2014). Fluorinated poly(propylenimine) dendrimers as gene vectors. Biomaterials.

[14] Wang F., Wang Y., Wang H., Shao N., Chen Y., Cheng Y. (2014). Synergistic effect of amino acids modified on dendrimer surface in gene delivery. Biomaterials.

[15] Wang M., Liu H., Li L., Cheng Y. (2014). A fluorinated dendrimer achieves excellent gene transfection efficacy at extremely low nitrogen to phosphorus ratios. Nat. Commun..

[16] Giljohann D.A., Seferos D.S., Prigodich A.E., Patel P.C., Mirkin C.A. (2009). Gene regulation with polyvalent siRNA-nanoparticle conjugates. J. Am. Chem. Soc..

[17] Kim S.T., Chompoosor A., Yeh Y.-C., Agasti S.S., Solfiell D.J., Rotello V.M. (2012). Dendronized gold nanoparticles for siRNA delivery. Small.

[18] Khan M., Ong Z.Y., Wiradharma N., Attia A.B.E., Yang Y.-Y. (2012). Advanced materials for co-delivery of drugs and genes in cancer therapy. Adv. Healthc. Mater..

[19] Jones C.H., Chen C.-K., Ravikrishnan A., Rane S., Pfeifer B.A. (2013). Overcoming nonviral gene delivery barriers: Perspective and future. Mol. Pharm..

[20] Fuhrhop J.-H., Wang T. (2004). Bolaamphiphiles. Chem. Rev..

[21] Zeng H., Johnson M.E., Oldenhuis N.J., Tiambeng T.N., Guan Z. (2015). Structure-based design of dendritic peptide bolaamphiphiles for siRNA delivery. ACS Cent. Sci..

[22] Jain N., Arntz Y., Goldschmidt V., Duportail G., Mély Y., Klymchenko A.S. (2010). New unsymmetrical bolaamphiphiles: Synthesis, assembly with DNA, and application for gene delivery. Bioconjugate Chem..

[23] Khan M., Ang C.Y., Wiradharma N., Yong L.-K., Liu S., Liu L., Gao S., Yang Y.-Y. (2012). Diaminododecane-based cationic bolaamphiphile as a non-viral gene delivery carrier. Biomaterials.

[24] Chen J.-X., Xu X.-D., Yang S., Yang J., Zhuo R.-X., Zhang X.-Z. (2013). Self-assembled bola-like amphiphilic peptides as viral-mimetic gene vectors for cancer cell targeted gene delivery. Macromol. Biosci..

[25] Eldredge A.C., Johnson M.E., Oldenhuis N.J., Guan Z. (2016). Focused library approach to discover discrete dipeptide bolaamphiphiles for siRNA delivery. Biomacromolecules.

[26] Huang Z., Zhang Y.-M., Cheng Q., Zhang J., Liu Y.-H., Wang B., Yu X.-Q. (2016). Structure-activity relationship studies of symmetrical cationic bolasomes as non-viral gene vectors. J. Mater. Chem. B.

[27] Fabio K., Gaucheron J., Di Giorgio C., Vierling P. (2003). Novel galactosylated polyamine bolaamphiphiles for gene delivery. Bioconjug. Chem..

[28] Wang B., Chen P., Zhang J., Chen X.-C., Liu Y.-H., Huang Z., Yu Q.-Y., Zhang J.-H., Zhang W., Wei X. (2017). Self-assembled core-shell-corona multifunctional non-viral vector with AIE property for efficient hepatocyte-targeting gene delivery. Polym. Chem..

[29] Ren W.X., Han J., Uhm S., Jang Y.J., Kang C., Kim J.-H., Kim J.S. (2015). Recent development of biotin conjugation in biological imaging, sensing, and target delivery. Chem. Commun..

[30] Cho D.Y., Cho H., Kwon K., Yu M., Lee E., Huh K.M., Lee D.H., Kang H.C. (2015). Triphenylphosphonium-conjugated poly(ε-caprolactone)-based self-assembled nanostructures as nanosized drugs and drug delivery carriers for mitochondria-targeting synergistic anticancer drug delivery. Adv. Funct. Mater..

[31] Sun J., Sheng R., Luo T., Wang Z., Li H., Cao A. (2016). Synthesis of diblock/statistical cationic glycopolymers with pendant galactose and lysine moieties: Gene delivery application and intracellular behaviors. J. Mater. Chem. B.

[32] Yi W.-J., Zhang Q.-F., Zhang J., Liu Q., Ren L., Chen Q.-M., Guo L., Yu X.-Q. (2014). Cyclen-based lipidic oligomers as potential gene delivery vehicles. Acta Biomater..

[33] Zhang Q.-F., Yu Q.-Y., Geng Y., Zhang J., Wu W.-X., Wang G., Gu Z., Yu X.-Q. (2014). Ring-opening polymerization for hyperbranched polycationic gene delivery vectors with excellent serum tolerance. ACS Appl. Mater. Interfaces.

[34] Zhu Y., Zhang J., Meng F., Deng C., Cheng R., Feijen J., Zhong Z. (2016). Crgd-functionalized reduction-sensitive shell-sheddable biodegradable micelles mediate enhanced doxorubicin delivery to human glioma xenografts in vivo. J. Control. Release.

[35] Koltover I., Salditt T., Rädler J.O., Safinya C.R. (1998). An inverted hexagonal phase of cationic liposome-DNA complexes related to DNA release and delivery. Science.

[36] Jain N., Goldschmidt V., Oncul S., Arntz Y., Duportail G., Mély Y., Klymchenko A.S. (2012). Lactose-ornithine bolaamphiphiles for efficient gene delivery in vitro. Int. J. Pharm..

[37] Liu Y., Reineke T.M. (2005). Hydroxyl stereochemistry and amine number within poly(glycoamidoamine)s affect intracellular DNA delivery. J. Am. Chem. Soc..

[38] Luan C.-R., Liu Y.-H., Zhang J., Yu Q.-Y., Huang Z., Wang B., Yu X.-Q. (2016). Low molecular weight oligomers with aromatic backbone as efficient nonviral gene vectors. ACS Appl. Mater. Interfaces.

[39] Zhang Q., Chen S., Zhuo R.-X., Zhang X.-Z., Cheng S.-X. (2010). Self-assembled terplexes for targeted gene delivery with improved transfection. Bioconjugate Chem..

[40] Nuraje N., Bai H., Su K. (2013). Bolaamphiphilic molecules: Assembly and applications. Prog. Polym. Sci..

