# Supplementary material for: Functionalized Asymmetric Bola-Type Amphiphiles for Efficient Gene and Drug Delivery
Source: Nanomaterials (Basel). 2018 Feb 13;8(2):115. doi: 10.3390/nano8020115 (PMC5853746; doi:10.3390/nano8020115)
Supplement: Supplementary file 1 [file nanomaterials-08-00115-s001.pdf]

Supplementary Files

Functionalized asymmetric bola-type amphiphiles for efficient gene and drug delivery

Zheng Huang, Dong-Mei Zhao, Xuan Deng, Ji Zhang*, Yi-Mei Zhang and Xiao-Qi Yu*

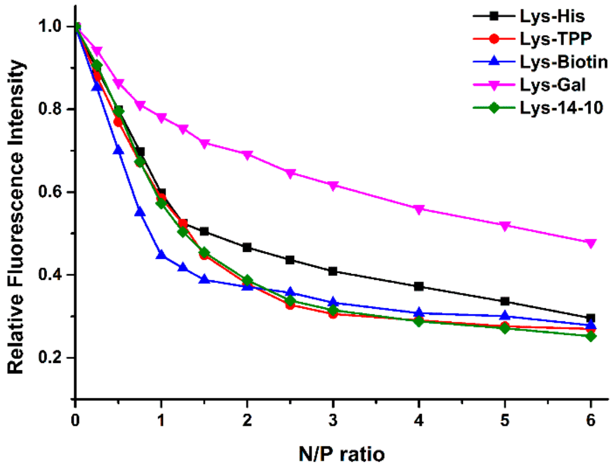

Figure S1. Fluorescent quenching assay of EB/DNA with the addition of bolasomes. The molar ratio of bolaamphiphile/DOPE was 1:1.

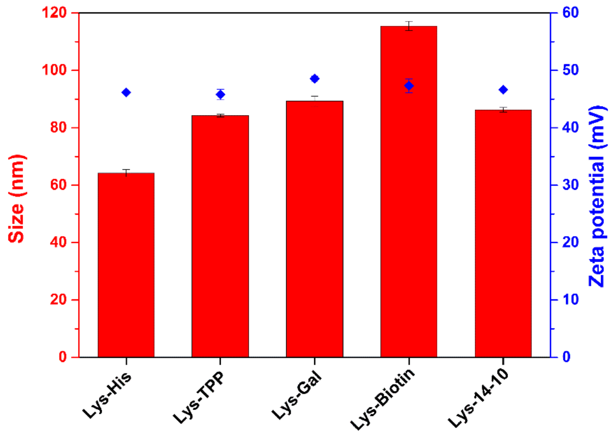

Figure S2. Mean particle sizes (columns) and zeta potentials (dots) of the five bolasomes with the DOPE/ bolaamphiphile ratio of 1:1 obtained by DLS at room temperature. Data represent mean ± SD (n = 3).

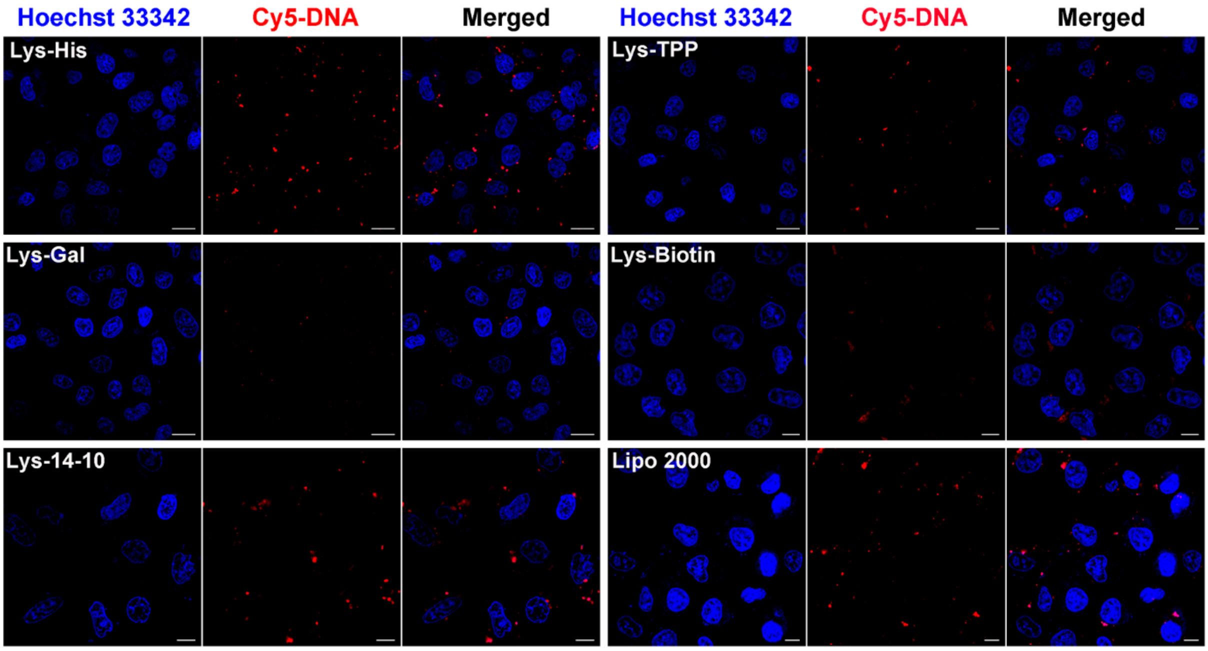

Figure S3. CLSM images of HeLa cells transfected with Cy5-labelled DNA by the bolaplexes at the optimal transfection N/P ratio. For each row, left: cell nuclei stained by Hoechst 33342 (blue); middle: Cy5-labelled pGL-3 DNA (red); right: merged image. Scale bar: 10 μm.

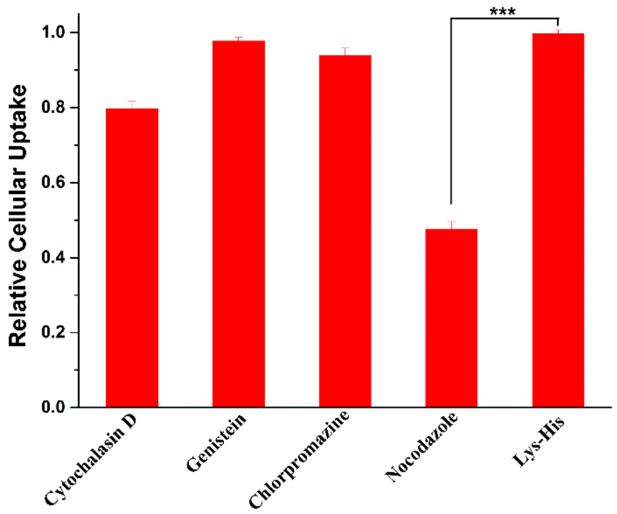

Figure S4. Relative cellular uptake of **Lys-His**/DNA bolaplexes at optimal transfection N/P ratio in HeLa cells in the presence of various endocytic inhibitors quantified by flow cytomety analysis. (***P < 0.001)

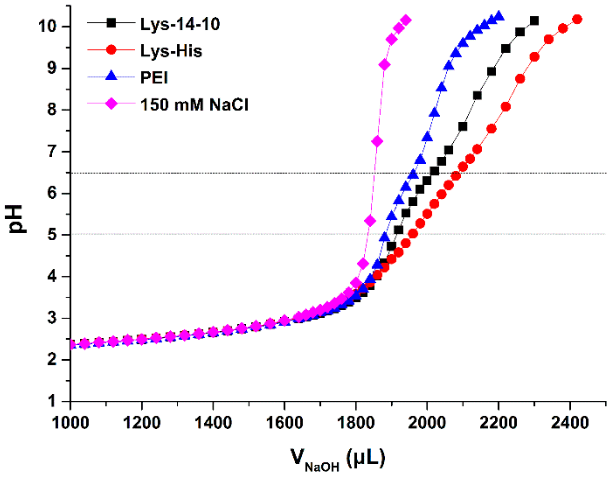

Figure S5. Acid–base titration profiles of **Lys-14-10**, **Lys-His**, 25 kDa PEI and 150 mM NaCl solutions. Bolaamphiphiles or PEI (0.050 mmol of amino groups) was first treated with 1 N HCl to adjust pH to 2.0, and then the solution pH was measured after each addition of 20 μL of 0.1 N NaOH.

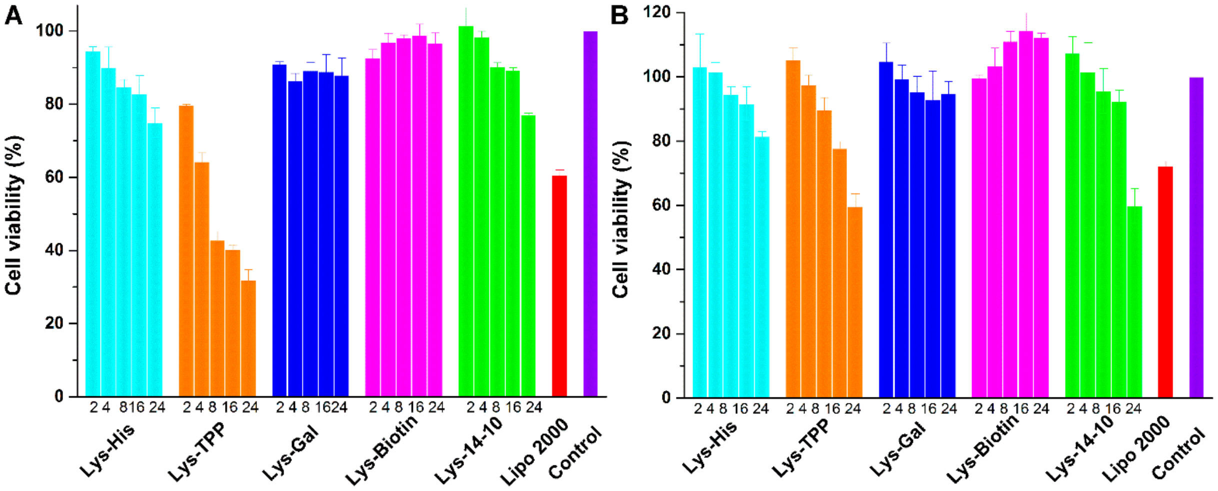

Figure S6. *In vitro* cytotoxicity of the bolaplexes at various N/P ratios in HepG-2 (A) and A549 cells (B) for a 24 h incubation. Data represent mean ± SD (n= 3)

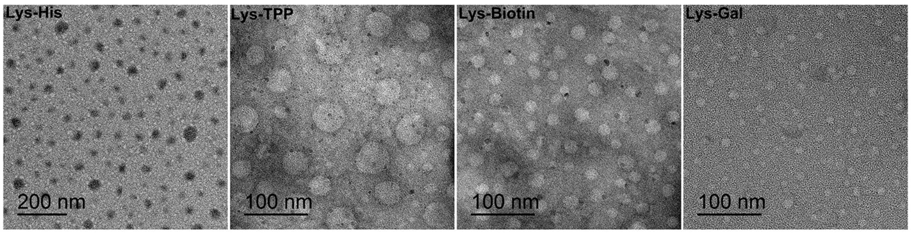

Figure S7. TEM images of the bolaamphiphile aggregates without DOPE

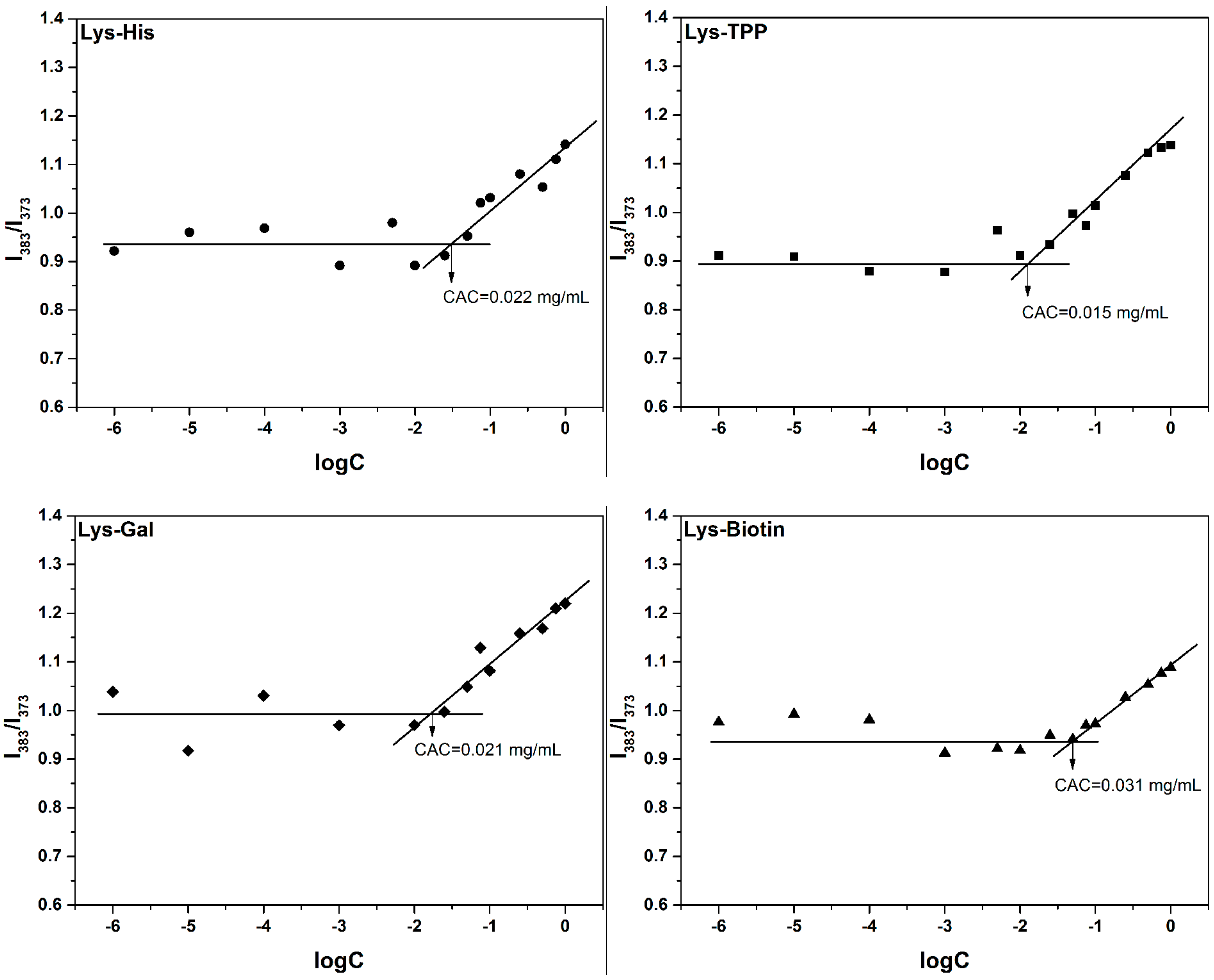

Figure S8. Plots of the intensity ratio I_{383}/I_{373} from the pyrene emission spectra *versus* the logarithm of the concentration for self-assembling aggregates in aqueous media from **Lys-His**, **Lys-TPP**, **Lys-Gal** and **Lys-Biotin**, respectively.

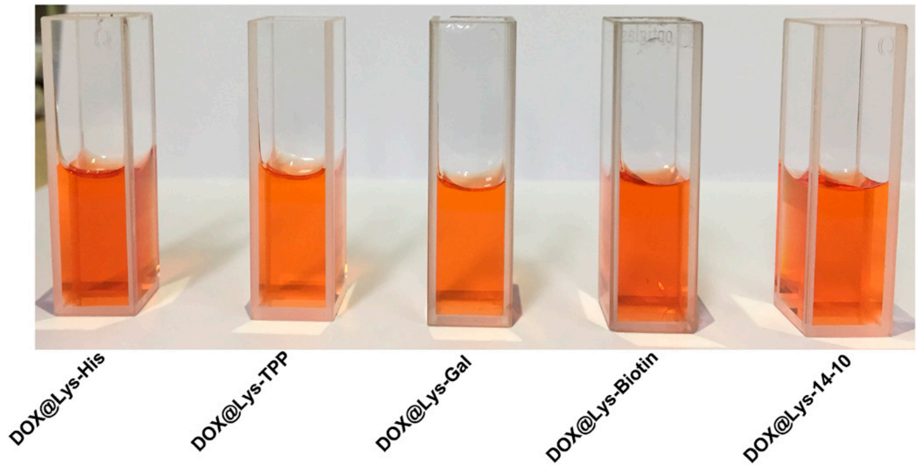

Figure S9. Aqueous drug-loaded bolaamphiphiles solutions after storage under 4ºC for 5 months.

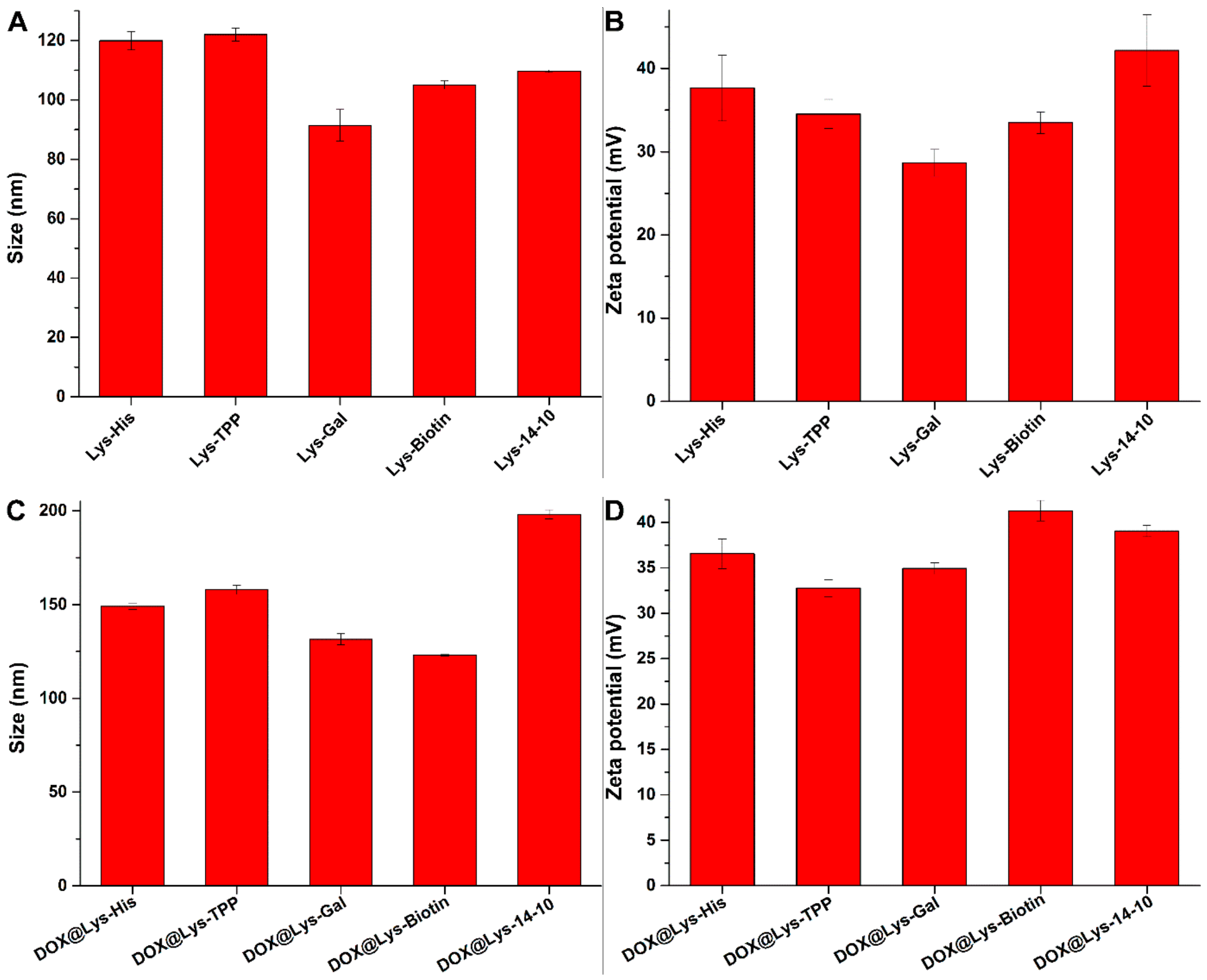

Figure S10. Mean particle sizes (A) and zeta-potentials (B) of drug-free bolaamphiphiles nanoparticles. Mean particle sizes (C) and zeta-potentials (D) of drug-loaded bolaamphiphiles nanoparticles measured by DLS at room temperature at a fixed angle (90°). Data represent mean ± SD (n = 3).

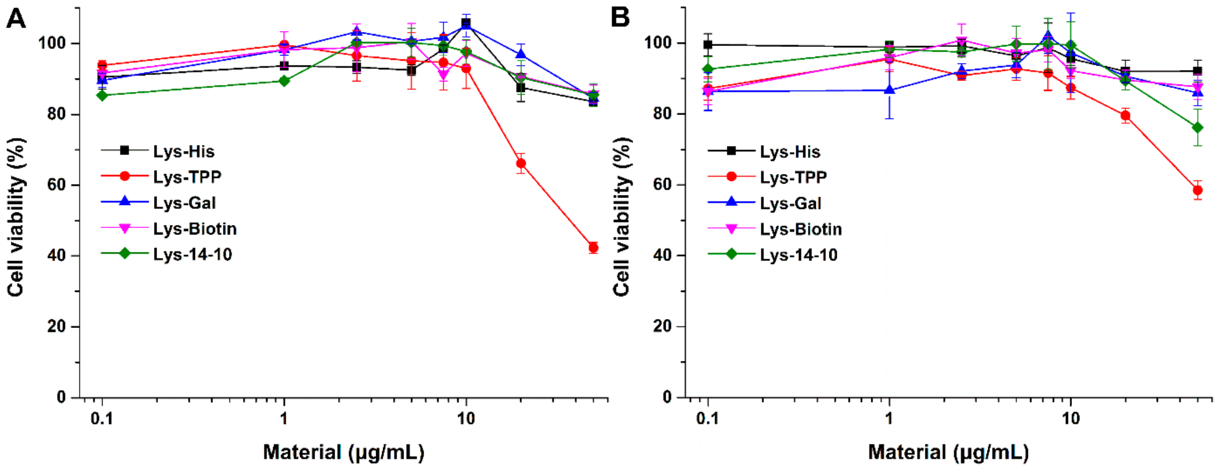

Figure S11. *In vitro* cytotoxicity of DOX-free bolaamphiphiles nanoparticles in HepG-2 (A) and HeLa (B) cells treated with various concentrations blank materials for 48 h. Data represent mean ± SD (n = 3).

Analysis data of Compound 2

Diol-14-10-OTBS: Colourless liquid, yield:65.2%.^{1}H NMR ($CDCl_3$, 400 MHz): δ (ppm) = 4.02 (t, 4H, *J*=8.0 Hz, COO-C*H*$_2$), 3.62-3.55 (m, 4H, HO-C*H*$_2$, Si-O-C*H*$_2$-), 2.26 (t, 4H, *J*=8.0 Hz, C*H*$_2$-OOC-), 1.58-1.23 (m, 52H, (C*H*$_2$)$_{26}$), 0.86 (s, 9H, -C(C*H*$_3$)$_3$), 0.02 (s, 6H, Si-C*H*$_3$). ^{13}C NMR ($CDCl_3$, 100 MHz): δ (ppm) = 174.02, 64.39, 64.35, 63.30, 63.29, 34.37, 32.84, 32.75, 29.55, 29.51, 29.48, 29.44, 29.41, 29.37, 29.26, 29.22, 29.19, 29.13, 28.60, 25.96, 25.90, 25.88, 25.75, 25.70, 25.62, 24.99, -5.28. HR-MS (ESI): $C_{40}H_{80}O_6SiNa$ [M+Na]$^+$, 707.5622, found: 707.5623.

Analysis data of Compound 3

2Boc-Lys-14-10-OH: White powder, yield: 53.8%. ^{1}H NMR ($CDCl_3$, 400 MHz): δ (ppm) = 4.63 (s, 1H, CONH-C*H*-), 4.06 (t, 2H, *J*=8.0 Hz, COO-C*H*$_2$), 4.00 (t, 4H, *J*=8.0 Hz, C*H*$_2$-OOC-), 3.58 (t, 2H, *J*=8.0 Hz, HO-C*H*$_2$,), 3.09-2.98 (m, 2H, CONH-C*H*$_2$), 2.24 (t, 4H, *J*=8.0 Hz, OOC-C*H*$_2$), 1.83-1.48 (m, 16H, CONH-CH$_2$CH$_2$CH$_2$-C*H*$_2$, CONH-CH$_2$-C*H*$_2$, COO-CH$_2$-C*H*$_2$,OOC-CH$_2$-C*H*$_2$, HO-CH$_2$-C*H*$_2$), 1.39 (s, 18H, Boc-*H*), 1.34-1.15 ((m, 40H, (C*H*$_2$)$_{20}$). ^{13}C NMR ($CDCl_3$, 100 MHz): δ (ppm) = 173.99, 172.84, 171.12, 156.01, 155.44, 79.71, 79.02, 65.37, 64.32, 62.87, 60.35, 53.24, 40.05, 34.34, 32.73, 32.36, 29.51, 29.46, 29.41, 29.38, 29.35, 29.22, 29.17, 29.12, 29.10, 28.58, 28.48, 28.37, 28.28, 25.86, 25.76, 25.69, 24.96. HR-MS (ESI): $C_{50}H_{94}N_2O_{11}Na$ [M+Na]$^+$, 921.6755, found: 921.6757.

Analysis data of target products

Lys-His: Flavescent ropy liquid, yield: 90.9%. ^{1}H NMR (CD_3OD, 400 MHz): δ (ppm) = 8.86 (s, 1H, imidazole-*H*), 7.45 (s, 1H, imidazole-*H*), 4.27-4.18 (m, 4H, Lys-COO-C*H*$_2$, His-COO-C*H*$_2$), 4.04 (t, 5H, *J*=8.0 Hz, C*H*$_2$-OOC-, imidazole-CH$_2$-C*H*), 3.44-3.33 (m, 2H, imidazole-C*H*$_2$), 3.31-3.27 (m, 1H, NH$_2$-(CH$_2$)$_4$-C*H*), 2.92 (t, 2H, *J*=8.0 Hz, NH$_2$-C*H*$_2$), 2.28 (t, 4H, *J*=8.0 Hz, OOC-C*H*$_2$), 1.99-1.86 (m, 2H, NH$_2$-(CH$_2$)$_3$-C*H*$_2$), 1.75-1.20 (m, 56H, (C*H*$_2$)$_{28}$). ^{13}C NMR (CD_3OD, 100 MHz): δ (ppm) = 174.24, 169.00, 167.71, 134.52, 127.29, 118.09, 66.68, 66.23, 64.04, 52.25, 51.53, 38.76, 33.69, 29.62, 29.25, 29.18, 29.17, 29.12, 28.96, 28.90, 28.75, 28.32, 28.13, 28.03, 26.59, 25.61, 25.44, 25.36, 25.23, 24.68, 21.67. HR-MS (ESI): $C_{46}H_{85}N_5O_8Na$ [M+Na]$^+$, 858.6296, found 858.6238.

Lys-TPP: Flavescent ropy liquid, yield: 93.4%. ^{1}H NMR (CD_3OD, 400 MHz): δ (ppm) = 7.91-7.70 (m, 15H, benzene-*H*), 4.27-4.19 (m, 2H, TPP-COO-C*H*$_2$), 4.07-3.97 (m, 6H, Lys-COO-C*H*$_2$, C*H*$_2$-OOC-), 3.45-3.41 (m, 1H, NH$_2$-(CH$_2$)$_4$-C*H*), 2.94 (t, 2H, *J*=8.0 Hz, NH$_2$-C*H*$_2$), 2.38 (t, 2H, *J*=8.0 Hz, (Ph)$_3$P-C*H*$_2$), 2.28 (t, 4H, *J*=8.0 Hz, OOC-C*H*$_2$), 2.00-1.90 (m, 2H, NH$_2$-(CH$_2$)$_3$-C*H*$_2$), 1.83 (t, 2H, *J*=8.0 Hz, (Ph)$_3$P-(CH$_2$)$_3$-C*H*$_2$), 1.76-1.20 (m, 58H, (C*H*$_2$)$_{29}$). ^{13}C NMR (CD_3OD, 100 MHz): δ (ppm) = 174.20, 173.24, 169.01, 134.90, 134.87, 133.43, 133.33, 130.18, 130.05, 118.84, 117.98, 114.92, 66.23, 64.23, 64.04, 61.56, 52.26, 38.78, 33.72, 32.48, 29.62, 29.26, 29.18, 29.16, 29.13, 28.97, 28.91, 28.75, 28.34, 28.29, 28.14, 26.58, 25.63, 25.57, 25.45, 25.43, 25.25, 24.70, 21.69. HR-MS (ESI): $C_{63}H_{101}N_2O_8P$ [M+H]$^{2+}$, 1044.7296, found: 522.3609(2).

Lys-Biotin: Flavescent ropy liquid, yield: 92.5%. ^{1}H NMR (CD_3OD, 400 MHz): δ (ppm) = 4.51-4.46 (m, 1H, CONH-C*H*), 4.32-4.27 (m, 1H, CONH-C*H*), 4.26-4.20 (m, 2H, Lys-COO-C*H*$_2$), 4.07-4.00 (m, 6H, C*H*$_2$-OOC-, Biotin-COO-C*H*$_2$), 3.31-3.21 (m, 1H, NH-C*H*), 3.22-3.16 (m, 1H, S-C*H*), 2.96-2.66 (m, 4H, NH$_2$-C*H*$_2$, S-C*H*$_2$), 2.35-2.25 (m, 6H, OOC-C*H*$_2$), 1.99-1.88 (m, 2H, NH$_2$-(CH$_2$)$_3$-C*H*$_2$), 1.78-1.20 (m, 62H, (C*H*$_2$)$_{31}$). ^{13}C NMR (CD_3OD, 100 MHz): δ (ppm) = 174.19, 173.99, 169.02, 164.66, 66.24, 64.15, 64.06, 61.97, 60.20, 55.58, 52.26, 39.66, 38.77, 33.74, 33.72, 33.47, 29.62, 29.27, 29.21, 29.19, 29.18, 28.98, 28.93, 28.91, 28.77, 28.35, 28.14, 28.09, 26.58, 25.66, 25.64, 25.45, 24.72, 24.71, 24.61, 21.67. HR-MS (ESI): $C_{50}H_{93}N_4O_9S$ [M+H]$^+$, 925.6663, found: 925.6654

Lys-Gal: Flavescent ropy liquid, yield: 91.5%. ^{1}H NMR (CD_3OD, 400 MHz): δ (ppm) = 4.36-4.31 (m, 1H, Galactose-*H*), 4.28-4.00 (m, 10H, -COO-C*H*$_2$), 3.82-3.40 (m, 5H, NH-C*H*, Galactose-*H*), 2.94 (t, 2H, *J*=8.0 Hz, NH_2-C*H*$_2$), 2.66-2.57 (m, 4H, Galactose-CH_2-OOC-C*H*$_2$), 2.28 (t, 4H, *J*=8.0 Hz, OOC-C*H*$_2$), 1.95-1.87 (m, 2H, NH_2-$(CH_2)_3$-C*H*$_2$), 1.77-1.22 (m, 56H, $(CH_2)_{28}$). ^{13}C NMR (CD_3OD, 100 MHz): δ (ppm) = 174.15, 172.74, 172.48, 169.06, 97.25, 92.79, 73.30, 72.44, 72.22, 69.56, 66.44, 66.23, 64.50, 64.09, 63.76, 63.56, 52.31, 38.82, 33.77, 33.59, 31.69, 29.60, 29.30, 29.22, 29.01, 28.96, 28.79, 28.54, 28.47, 28.37, 28.32, 28.14, 27.29, 26.55, 25.69, 25.62, 25.46, 24.74, 22.94, 21.67. HR-MS (ESI): $C_{50}H_{93}N_2O_{15}$ $[M+H]^+$, 961.6576, found: 961.6559.
